# Découverte de Foyers de Poliovirus de Type-2 Dérivé du Vaccin Antipoliomyélitique Oral en République Centrafricaine en 2019

**DOI:** 10.48327/mtsibulletin.2021.114

**Published:** 2021-06-20

**Authors:** E. Kalthan, I. Gouandjika-Vasilache, R. Mbailao, J.W. Doté, M.N. Kossone, M. Gbangai

**Affiliations:** 1 Ministère de la santé et de la population, direction de la surveillance épidémiologique et de gestion d'urgence en santé publique, République centrafricaine; 2 Institut Pasteur de Bangui, laboratoire régional de référence OMS pour la poliomyélite en Afrique, République centrafricaine; 3 Ministère de la santé et de la population, direction générale de l'épidémiologie et de lutte contre les maladies, République centrafricaine; 4 Ministère de la santé et de la population, district sanitaire de Bambari, République centrafricaine; 5 Ministère de la santé et de la population, service de la vaccination, République centrafricaine

**Keywords:** Paralysie flasque aiguë, Vaccination, PVDVc2, VPI, Vaccin antipoliomyélitique oral, Vaccin polio injectable, Couverture vaccinale, Bambari, Bimbo, Mbaiki, Bangui, Kemo, Berberati, Bangassou, Bocaranga, Nola, République centrafricaine, Afrique subsaharienne, Acute flaccid paralysis, Vaccination, PVDVc2, IPV, Oral polio vaccine, Injectable polio vaccine, Vaccination coverage, Bambari, Bimbo, Mbaiki, Bangui, Kemo, Berberati, Bangassou, Bocaranga, Nola, Central African Republic, Subsaharan Africa

## Abstract

**Objectif:**

En 2019, la République centrafricaine a découvert des foyers de poliovirus de type 2 dérivé vaccinal circulant (PVDV2c). L'objectif de ce travail est de décrire l'état vaccinal des enfants paralysés par le PVDV2c et leurs contacts et d'évaluer la circulation de cette souche chez ces contacts.

**Patients et Méthode:**

Il s'agit d'une étude rétrospective et descriptive. La population d'étude est constituée des enfants atteints de paralysie flasque aiguë (PFA) et de leurs contacts. Nous avons inclus les enfants paralysés dont les résultats de séquençage ont montré la présence du PVDV2c.

**Résultats:**

Au total, 21 enfants paralysés par le PVDVc et 64 contacts ont été enrôlés dans cette étude. Quatorze enfants paralysés sur 21 (66%) avaient reçu au moins une dose de vaccin antipoliomyélitique oral (VPO) bivalent contre 36 sur 64 (57%) chez les contacts (différence non significative). Parmi les malades vaccinés, 7 avaient reçu moins de trois doses. Pour le vaccin polio injectable (VPI), la couverture vaccinale chez les malades comme pour les contacts était de 33%.

La proportion des enfants ayant reçu à la fois des doses de VPO et de VPI était de 33% chez les malades et 25% chez les contacts. Les contacts porteurs de VDPV2 ont été vaccinés par le VPO et VPI, respectivement 55% et 27%. Le VDPV2 et le Sabin 2 ont également été trouvés dans les selles des contacts, respectivement 34% et 9%.

**Conclusion:**

L'insuffisance de vaccination en VPO et VPI pendant plusieurs années a des conséquences graves par la survenue de virus dérivé du vaccin et responsable de paralysie à vie. Pour arrêter l'épidémie de VDPVc, il faut renforcer la couverture vaccinale par une campagne de masse couvrant tous les districts en même temps.

## Abstract (en)

**Objective:**

In 2019, the Central African Republic identified foci of circulating vaccine-derived poliovirus 2 (PVDV2c). The objective of this work is to describe the vaccination status of children paralyzed by PVDV2c and their contacts and to assess the circulation of this strain in these contacts.

**Patients and method:**

The study population of this retrospective survey consists of children with acute flaccid paralysis (AFP) and their contacts. We included paralyzed children whose sequencing results showed the presence of PVDV2c.

**Results:**

A total of 21 children paralyzed by PVDVc and 64 contacts were enrolled in the survey. Fourteen out of 21 children who are paralyzed (66%) received at least one dose of bivalent oral polio vaccine (OPV) compared to 36 out of 64 contacts (57%, non-significant difference). Of the vaccinated patients, 7 had received less than three doses. For the injectable polio vaccine (IPV), vaccination coverage for both patients and contacts was 33%.

The proportion of children who received both doses of OPV and IPV was 33% among patients and 25% in contacts. Contacts with VDPV2 were vaccinated with OPV and IPV, respectively 55 and 27%. VDPV2 and Sabin 2 were also found in contact stools, 34% and 9% respectively.

**Conclusion:**

The absence or inadequacy of IPV vaccination has a serious impact on children by the occurrence of virus derived from the vaccine responsible for life-old paralysis. Protecting children from poliomyelitis requires a combination of a good cold chain, multiple doses and adherence to the vaccine schedule.

## Introduction

La poliomyélite est une maladie à prévention vaccinale grâce à deux vaccins dont un vaccin injectable inactivé (VPI) et un vaccin oral (VPO) constitué de souches de poliovirus atténuées des trois sérotypes [2]. Le VPO, à cause de sa facilité dans la vaccination de masse par rapport au VPI, est utilisé extensivement pour éradiquer la maladie [7]

La République centrafricaine (RCA) s'est engagée dans l'initiative mondiale d'éradication de la poliomyélite. Elle a intégré le VPO dans le programme de vaccination de routine. Le dernier cas de poliovirus sauvage (PVS) autochtone date de décembre 1999 et le dernier cas de poliovirus sauvage importé remonte à décembre 2011. Trois ans avant l'éradication officielle, mondiale, du poliovirus de type 2 en septembre 2015, le pays n'a plus détecté de poliovirus sauvage.

Du fait de la recrudescence des paralysies post-vaccinales dues au poliovirus de type 2, le VPO trivalent a été retiré du programme de vaccination de routine en RCA en avril 2016. Le vaccin bivalent contenant les souches de poliovirus de type 1 et 3 a été introduit en mai 2016. Avant le premier anniversaire, l'enfant doit recevoir 4 doses de VPO bivalent.

Le vaccin polio injectable (VPI) à souche désactivée, dans le but de retirer progressivement le VPO, a été introduit en juin 2015 dans le service de vaccination de routine. Il est administré à partir de la 14e semaine.

Dans le contexte des crises récurrentes politico-militaires touchant le pays depuis des décennies, les services de santé et particulièrement de la vaccination dysfonctionnent fréquemment. La couverture vaccinale est restée faible pendant des années.

Le système de surveillance de la paralysie flasque aiguë (PFA) s'inscrit dans le cadre global de la surveillance intégrée des maladies et riposte (SIMR). La surveillance active de la PFA est basée sur la visite périodique des sites classés en quatre catégories de priorité selon l'épidémiologie de la zone. Classée parmi les maladies à déclaration obligatoire et immédiate, la surveillance des cas de PFA est rapportée suivant un rythme hebdomadaire des formations sanitaires vers le niveau hiérarchique supérieur pour analyse. Chaque PFA et ses contacts font l'objet de prélèvement de selles à acheminer au laboratoire de référence en observant les critères de performance de la surveillance. Les cas où les échantillons sont declares inadéquats font l'objet d'examen de suivi entre 60 et 90 jours.

En vue d'atteindre l'objectif d'éradication de la poliomyélite, quatre stratégies ont été définies. Il s'agit d'atteindre une couverture vaccinale de routine d'au moins 80%, de renforcer la surveillance de PFA, d'organiser des activités de vaccination supplémentaires et des campagnes de rattrapage/riposte localisées.

Le pays a notifié son premier cas de poliovirus de type 2 dérivé vaccinal circulant (PVDV2c) en mai 2019 malgré le remplacement du VPO trivalent par le bivalent et l'introduction du VPI.

L'objectif de ce travail est de décrire l'état vaccinal des enfants paralysés par le PVDV2c et leurs contacts et d'évaluer la circulation de cette souche chez ces contacts.

## Historique

En mai 2019, un poliovirus dérivé de poliovirus vaccinal de type 2 (différenciation intra-typique et séquençage) a été découvert à l'Institut Pasteur de Bangui, laboratoire régional de référence de l'OMS pour la poliomyélite en Afrique [9]. Une investigation a été menée du 15 au 20 mai 2019 dans le district sanitaire de Bambari, lieu de résidence de ce premier cas. La ville de Bambari compte dix sites de personnes déplacées fuyant les conflits armés. Le site de l'Élevage est le lieu de résidence de l'enfant atteint de PFA chez qui le poliovirus a été isolé. C'est un camp ouvert utilisé par les Peulhs en provenance des villes centrafricaines, du Tchad, du Niger et du Cameroun. Il comptait environ 18000 personnes en 2014 et 9900 en 2019. Les conditions d'hygiène y étaient très précaires.

Dix échantillons de selles ont été prélevés chez des enfants contacts du cas index. Les résultats de séquençage réalisé à Paris et à Johannesburg, validés par le CDC Atlanta, ont confirmé la présence du poliovirus dérivé du type 2 le 29 mai 2019.

**Figure 1:**
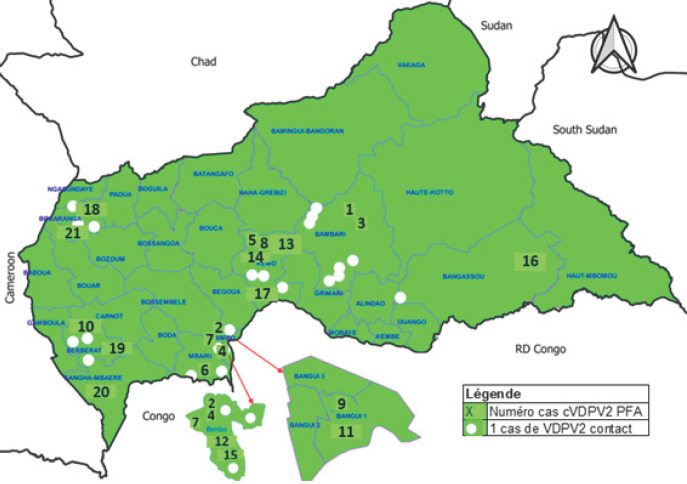
Distribution chronologique des cas de PVDV2c par district et par semaine épidémiologique en RCA en 2019 Chronologic distribution of PVDV2 cases by district and epidemiological weeks in CAR map in 2019

La déclaration de la présence de ce virus suite à la détection du premier cas, le renforcement de la surveillance épidémiologique et l'appui des consultants internationaux pour la riposte ont permis de détecter d'autres cas de VDPV2c respectivement à Bimbo, Mbaiki, Bangui, Kemo, Berberati, Bangassou, Bocaranga et Nola (Fig. 1). Quatre nouveaux cas ont été détectés en 2020.

## Patients et Méthodes

Il s'agit d'une étude rétrospective à visée descriptive portant sur l'ensemble des cas avec PFA pour lesquels un poliovirus dérivé d'une souche circulante du VPO a été mis en évidence dans les selles du 3 mai au 29 décembre 2019, ainsi que sur les sujets contacts de ces cas. Pour chaque cas de PFA due à un PVDVc, deux à trois prélèvements de selles ont été réalisés chez les enfants contacts. Un enfant contact est un enfant vivant sous le même toit que l'enfant paralysé ou vivant au voisinage direct de ce cas.

Les variables étudiées étaient l'âge, le sexe, le statut vaccinal, le lieu de résidence et les signes cliniques. Les données ont été saisies sur Excel 2007 et analysées avec Epi Info 7. La comparaison des données a été faite grâce au test de khi^2^ avec un seuil de signification inférieure à 0,05.

## Résultats

**Figure 2:**
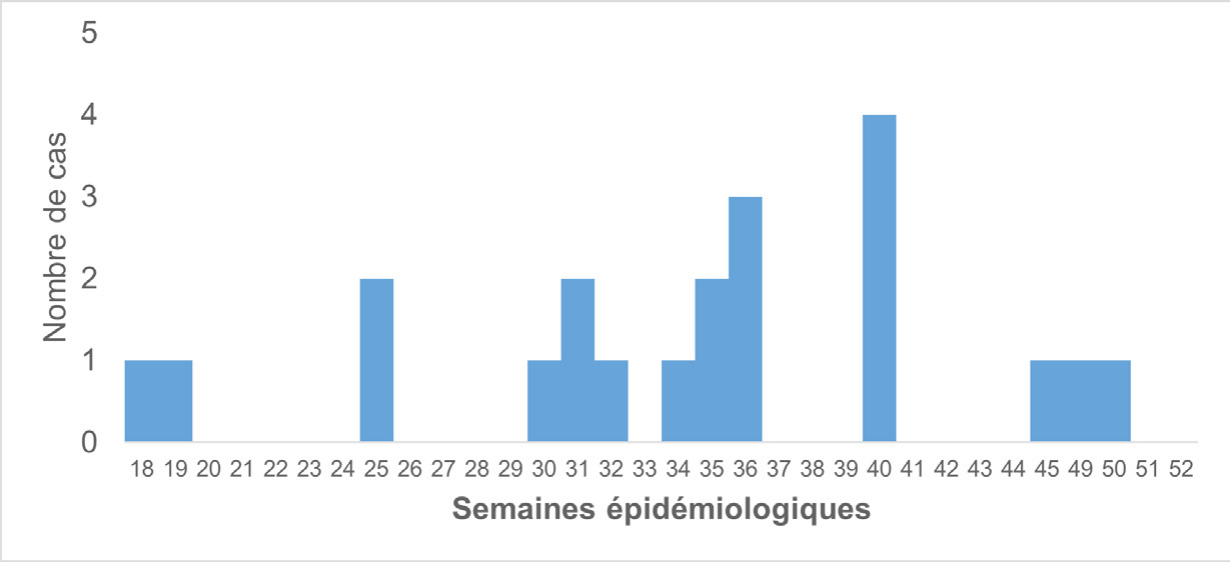
Photo du cas index de VDPV2 au site des déplacés Elevage à Bambari, RCA The index case of VDPV2 in the spot Elevage of displaced people in Bambari, CAR

Au total, 21 enfants atteints de PFA et 64 contacts des 8 sites contrôlés chez qui le PVDV2c a été isolé et confirmé par le séquençage, ont été enrôlés (Fig. 1). Le cas index était une fillette de trois ans présentant une paralysie de la jambe droite (Fig. 2). Elle n'avait reçu aucune dose de VPO depuis la naissance. La malade n'avait pas effectué de déplacement avec ses parents dans les 30 jours précédant la paralysie.

**Figure 3:**
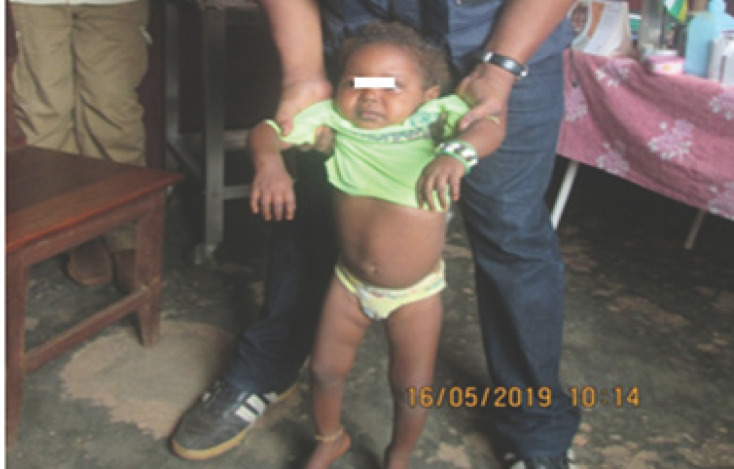
Histogramme de cas de PVDV2c circulant détectés en RCA en 2019 Histogram of circulating PVDV2 cases detected in CAR in 2019

L'âge moyen des malades et des contacts était respectivement de 24 [Écart Type = 0,96] et 36 mois [ET = 1,56] avec les extrêmes (7 mois - 5 ans et 2 mois – 8 ans). Le premier cas était détecté à la semaine épidémiologique numéro 18 de 2019. Le pic était observé à la semaine 40 (Fig. 3). Les enfants de sexe masculin représentaient 52% des cas. La fièvre était présente chez 16 enfants sur 21. La paralysie a été observée sur les deux jambes dans 12 cas sur 21 (Tableau I). Quatorze enfants paralysés sur 21 avaient reçu au moins une dose de VPO contre 36 sur 64 chez les contacts, différence non significative (Tableau II). La couverture vaccinale chez les malades et les contacts par le VPI était de 33%. La proportion des enfants ayant reçu à la fois des doses de VPO et de VPI était de 33% chez les malades et 25% chez les contacts. Les contacts porteurs de VDPV2 ont été vaccinés par le VPO et VPI respectivement à 55% et 27%. Le PVDV2c et le Sabin 2 ont été trouvés respectivement dans 34% et 9% des selles des contacts (Tableau II).

**Tableau I:** Situation détaillée des enfants paralysés par le PVDV2c Spatial distribution of the children suffering paralysis by PVDV2c

N° EPID	Districts	Âge	Sexe	Localisation paralysie	Doses de VPO reçu	Doses de VPI reçu
58	BAMBARI	3	F	JD	0	0
62	BIMBO	3	F	JGD	0	0
79	BAMBARI	2	M	JGD	2	0
81	BIMBO	2	F	JD	1	1
103	KEMO	2	M	JGD	4	1
108	MBAIKI	2	F	JG	1	Inconnu
115	BIMBO	3	F	JG	2	0
124	KEMO	1	M	JGD	4	0
141	BANGUI I	2	M	JG	Inconnu	1
147	BERBERATI	2	F	JGD	0	0
152	BANGUI I	5	M	JG	0	0
154	BIMBO	2	M	JG	1	0
155	KEMO	2	F	JGD	5	Inconnu
170	KEMO	1	M	JGD	3	0
172	BIMBO	1	M	JGD	6	1
175	BANGASSOU	2	M	JGD	1	1
176	KEMO	1	M	JGD	7	1
190	BOCARANGA-KOUI	0	M	JGD	0	Inconnu
220	BERBERATI	2	F	JGD	4	0
222	SANGHA-MBAERE	1	F	JG	6	1
223	BOCARANGA-KOUI	3	F	JG	0	Inconnu

JG = jambe gauche; JD = jambe droite; JGD = jambe gauche et droite

**Tableau II:** Répartition des cas et des contacts de PVDV2c selon le statut vaccinal Distribution of cases and contacts of PVDV2c by vaccine status

Statut vaccinal	Cas de PFA avec PVDP2c positifs		Enfants contacts de PFA	
	VPO	VPI	VPO	VPI
	Nombre	Nombre	Nombre	Nombre
Vaccinés	14	7	36	21
Non vaccinés	5	10	16	29
Inconnu	2	4	12	14
**Total**	**21**	**21**	**64**	**64**

**Tableau III:** Recherche de poliovirus dans les selles des cas contacts Poliovirus results in the stolls of contact cases

Résultats	Nombre
**Négatifs de poliovirus**	23
**Entérovirus Non Polio (NPENT)**	13
**Sabin de type 2**	6
**VDPV2**	22
**Total**	**64**

## Discussion

Pour garantir la validité des données collectées, plusieurs mesures ont été mises en place. La fiche d'enquête de cas de PFA a été remplie lors de la consultation. Une validation systématique à domicile du cas par un personnel plus qualifié assisté d'un expert de l'OMS a été réalisée dans les sept jours. Enfin, le support de validation du cas de PFA contient les mêmes variables que la fiche d'enquête. A cette occasion, une éventuelle erreur suite à la déclaration de la mère de l'enfant était corrigée par la vérification du carnet de vaccination ou d'autres documents.

Le virus PVDV2 a été détecté à Bambari puis dans les districts de santé de Bimbo, Mbaiki, Bangui 1, Kemo, Berberati, Bangassou, Bocaranga-Koui et Sangha-Mbaere. Les mauvaises conditions d'hygiène, les déplacements et regroupements de population ont pu favoriser sa diffusion. Cette diffusion du virus a été observée également dans certains pays africains comme la RDC où le PVDV2c a été mis en évidence chez 29 enfants [1] ainsi qu'au Nigéria de 2005 à 2012 avec presque 400 cas de PFA [2, 11].

La vaccination de routine d'un enfant en RCA devrait être accomplie avant son premier anniversaire. Les enfants de notre série, avec un âge moyen de 24 mois, n'avaient pas été vaccinés dans 23% des cas. Presque la moitié des enfants vaccinés avaient reçu seulement une dose de VPO. Bien que la couverture vaccinale de routine en VPO3 soit croissante de 2015 (47%) à 2019 (81%) sur le plan national, il y a des districts avec une très faible activité vaccinale parmi lequel le district de Bambari. Une couverture vaccinale inférieure à 80% n'empêche pas la circulation du poliovirus.

La couverture nationale en VPI est passée de 42 à 81% de 2017 à 2019 avec des disparités entre les districts. En 2018 et 2019, la proportion des districts avec couverture vaccinale en VPI supérieure ou égale à 80% était respectivement 45 et 54%. Mais cette couverture vaccinale chez les malades et les contacts observés dans cette étude était inférieure à 50%.

La différence entre le statut vaccinal des malades et des contacts n'était pas significative. A priori, les enfants vaccinés au VPO/VPI ne devraient pas attraper la poliomyélite due au virus d'origine vaccinale (type 2) ou sauvage. Cet échec vaccinal pourrait être expliqué de plusieurs manières. La plupart des enfants vaccinés n'avaient pas terminé leurs doses de VPO. Nos résultats corroborent ceux de Sylla et collaborateurs dont 75% des malades avaient un statut vaccinal incomplet [9]. Dans ces conditions, la protection est faible. Pour être totalement efficace, le VPO doit être administré plusieurs fois [8]. Il est conçu pour être administré à multiples doses en vue de conférer une protection complète. Dans les régions tropicales, plusieurs doses (parfois plus de dix) sont nécessaires pour protéger totalement un enfant [8].

La confirmation de VDPV2 chez les enfants paralytiques a été renforcée, durant la même période, par la détection de 9 cas de PVDV2c circulant par la surveillance environnementale dans les mêmes districts (Bambari et Bangui-1) et dans le nouveau district de Bangui-2. Nos données corroborent celles de la RDC en ce qui concerne la circulation environnementale du PVDV2c [1]. Le PVDV2c est présent chez un tiers des cas contacts de cette étude. Associé à un taux de paralysie faible, il peut circuler de manière silencieuse [3, 5, 6].

Les souches Sabin 2 ont été trouvées chez certains contacts. Ces souches sont excrétées par les enfants après la vaccination avec le VPO de type 2. Cette présence en RCA, pays ayant retiré le VPO2, pourrait s'expliquer par l'arrivée d'un enfant vacciné avec le VPO2 probablement dans un pays voisin en riposte contre le PVDV2c. Le nombre de Sabin 2 avait augmenté après les campagnes de riposte organisées en RCA. Selon Delpeyroux, les PVDV2c malgaches pourraient être issus d'événements de recombinaison entre la souche mutée du Sabin 2 du VPO et des entérovirus non-polio du groupe C [2]. L'étude de rares cas de paralysies flasques aiguës temporellement associés à la vaccination a fortement mis en cause les souches Sabin qui avaient récupéré leur neuro-virulence lors de leur réplication dans l'intestin des personnes vaccinées [10].

Après la déclaration officielle de l'épidémie comme urgence de santé publique de portée nationale le 30 mai 2019 par le Ministre de la Santé et de la Population, une analyse des risques a été faite. Une campagne de riposte vaccinale utilisant le VPO2 à deux tours avec ratissage a été organisée. La première campagne ou « Round 0 » s'est déroulée du 16 au 19 juin 2019 dans les localités couvrant les districts de Bambari, Bimbo et les districts voisins. Le 2e et le 3e passage ont couvert 21 districts à risque élevé de poliomyélite. Dans la littérature, l'organisation de vaccination de riposte peut stopper la diffusion du virus [1, 4]. Cependant, malgré la bonne couverture vaccinale confirmée par l'enquête post-riposte, cette diffusion ne s'est pas arrêtée, entraînant l'organisation de cinq autres campagnes de vaccination de masse.

## Conclusion

L'insuffisance de vaccination contre la poliomyélite en RCA depuis plusieurs années est associée à la survenue de virus dérivé du vaccin, responsable de paralysie à vie. Le déplacement et le regroupement de populations peuvent accélérer sa diffusion, souvent silencieuse. La couverture vaccinale doit être renforcée dans l'ensemble du pays et s'appuyer sur des actions simultanées dans tous ses districts sanitaires.

## Remerciements

Les auteurs remercient Mr Ouedraogo Salfo pour avoir fourni la base des données et Mr Pamatika Christian Mocler pour la relecture de l'article. Nous remercions également le personnel technique du Laboratoire de référence OMS (Institut Pasteur de Bangui) pour la poliomyélite en Afrique pour la qualité des résultats de laboratoire.

## Conflits D'intérêts

Les auteurs ne déclarent aucun conflit d'intérêt.
